# Understanding violations of Gricean maxims in preschoolers and adults

**DOI:** 10.3389/fpsyg.2015.00901

**Published:** 2015-07-02

**Authors:** Mako Okanda, Kosuke Asada, Yusuke Moriguchi, Shoji Itakura

**Affiliations:** ^1^Department of Psychology, Otemon Gakuin UniversityOsaka, Japan; ^2^Research Center for Advanced Science and Technology, The University of TokyoTokyo, Japan; ^3^Department of School Education, Joetsu University of EducationJoetsu, Japan; ^4^Japan Science and Technology AgencyPRESTO/Sakigake, Tokyo, Japan; ^5^Department of Psychology, Graduate School of Letters, Kyoto UniversityKyoto, Japan

**Keywords:** cognitive development, conversational process, language development, Gricean maxims, pragmatics

## Abstract

This study used a revised Conversational Violations Test to examine Gricean maxim violations in 4- to 6-year-old Japanese children and adults. Participants' understanding of the following maxims was assessed: be informative (first maxim of quantity), avoid redundancy (second maxim of quantity), be truthful (maxim of quality), be relevant (maxim of relation), avoid ambiguity (second maxim of manner), and be polite (maxim of politeness). Sensitivity to violations of Gricean maxims increased with age: 4-year-olds' understanding of maxims was near chance, 5-year-olds understood some maxims (first maxim of quantity and maxims of quality, relation, and manner), and 6-year-olds and adults understood all maxims. Preschoolers acquired the maxim of relation first and had the greatest difficulty understanding the second maxim of quantity. Children and adults differed in their comprehension of the maxim of politeness. The development of the pragmatic understanding of Gricean maxims and implications for the construction of developmental tasks from early childhood to adulthood are discussed.

## Introduction

### Background of this study

Pragmatic abilities are key to interpreting other people's utterances and responding to them appropriately. These abilities are acquired during childhood and encompass a variety of skills, such as reading the minds of others, communicating with them according to their statements, and understanding conversational rules. Grice (1975) identified rules for conversation, known as conversational maxims, based on which people interpret others' utterances. According to Grice, people assume that normal conversation should follow these rules, and they try to infer the underlying meaning of utterances in which the maxims are violated. For example, someone who enters a dirty room and says, “What a beautiful room this is!” violates the rule that one should tell the truth. A listener who can detect this violation may infer the speaker's underlying meaning to be sarcasm. Understanding conversational rules is therefore fundamental for smooth communication, and conversational rules that are shared in society may contribute to mutual understanding during conversation. However, young children may have difficulty understanding some of these conversational rules. Knowing the age at which children begin to understand these rules, as well as the specific rules that are understood earlier or later, can contribute to the understanding of characteristics of children's conversation and identification of the most effective ways for adults (teachers, court judges, researchers, and so on) to talk to children.

Despite the importance of the Gricean maxims, few empirical studies have examined children's (and adults') understanding of them, and the results have been mixed, because tasks and children's age groups varied between studies. Further, only a few maxims have been examined; the maxim of manner has yet to be tested despite its assumed importance.

### Inconsistent results of previous studies

According to Grice (1975), the cooperative principle makes several requirements of speakers. They must “avoid providing less or more information than is required for the current purposes of the exchange” (first and second maxims of quantity), “be truthful and avoid saying something lacking adequate evidence” (maxim of quality), “be relevant” (maxim of relation), “avoid obscurity of expression, avoid ambiguity, be brief (avoid unnecessary prolixity), and be orderly” (four maxims of manner). An additional maxim of politeness (“be polite”) can also be added to these four maxims.

Several studies have examined preschoolers' and school-age children's understanding of the Gricean maxims (Ackerman, 1981; Conti and Camras, 1984; Axia and Baroni, 1985; Surian et al., 1996; Eskritt et al., 2008; Siegal et al., 2009, 2010; Vázquez et al., 2013), with mixed results. Some studies have established that children are able to understand and explain violations of the Gricean maxims (i.e., quantity and relation) only after they have started formal schooling (Ackerman, 1981; Conti and Camras, 1984; Axia and Baroni, 1985), but other studies have found that even younger children showed some understanding of the maxims (Eskritt et al., 2008; Gillis and Nilsen, 2013; Vázquez et al., 2013).

These studies have used a variety of tasks to gauge children's understanding. In a story-telling task, children were told several short stories that included question-exchange conversations with two different endings (i.e., one respondent violated one of the Gricean maxims, while the other gave an appropriate response) and were asked to choose a funny or silly response (Conti and Camras, 1984). School-aged children were able to perform this task, showing an understanding of Gricean maxims. In another task, children were asked to assign an utterance to one of two imaginary characters, “Honest Alice,” a Gricean follower, or “Saucy Sally,” a Gricean flouter (Ackerman, 1981). School-aged children, but not preschoolers, were able to discriminate the utterances, and only 8- to 9-year-olds were able to explain the violations of conversational rules. Axia and Baroni (1985) examined 5-, 7-, and 9-year-olds' understanding of politeness in social situation settings by recording and analyzing the children's responses to adults who showed accounted or unaccounted refusal and the children's response to someone's request to others (adult vs. child). Only the 9-year-olds demonstrated this understanding.

However, 2-year-olds may also have some awareness of Gricean maxims (Pellegrini et al., 1987; Dunham et al., 2000). Eskritt et al. (2008) developed a selective trust paradigm to examine 3–5-year-olds' understanding of the first maxim of quantity (providing inadequate information) and the maxims of quality and relation. Children were asked to find a sticker that was hidden beneath one of four different-colored cups and could seek help from one of two puppets, a Gricean follower or a Gricean flouter. For example, if the sticker was hidden under the blue cup, the Gricean follower would say, “It is under the blue cup,” but the Gricean flouter (who violated the maxim of relation) would say, “I like cups.” Children were best able to understand violations of the maxim of relation. In addition, 3-year-olds never performed above chance when a puppet violated the first maxim of quantity. Therefore, Eskritt et al. (2008) indicated that the maxim of relation may be the easiest one to understand (see also Surian et al., 1996, 2010). A similar study, also using a selective trust paradigm, confirmed that 4- and 6-year-olds were able to learn new words from a good conversation partner (a trustworthy adult), rather than from bad partner (an untruthful adult who violated the maxim of quality or relation (Vázquez et al., 2013). Furthermore, 4- to 7-year-olds preferred a speaker who gave sufficient information over a speaker who gave insufficient information (i.e., a speaker who violated the first maxim of quantity) (Gillis and Nilsen, 2013).

### Conversational violations test (CVT)

Preschoolers are likely to show their awareness of Gricean maxims in their own speech (Pellegrini et al., 1987) and during game-based tasks (Eskritt et al., 2008; Gillis and Nilsen, 2013; Vázquez et al., 2013); however, they perform poorly on observational tasks in which they must observe the utterances of others (Ackerman, 1981; Conti and Camras, 1984). Children cannot be involved in conversations during observational tasks as they are in game-based tasks, and this may result in poor performance in preschoolers. However, there is an observational task that can measure preschoolers' understanding of Gricean maxim violations. The Conversational Violations Test (CVT) contains simple question-answer conversations, with two alternative answers to one question. Specifically, children are presented with a video (or a tape, see Surian et al., 1996) in which three puppets carry on short, question-answer conversations (20 or 25 conversations in total). In this task, one puppet asks a question of two other puppets. One of the puppets gives an appropriate answer, and the other gives an inappropriate answer that violates one of the Gricean maxims. Children must then decide which one of the puppets answered inappropriately. For example, the puppet may ask, “What games do you know?” One puppet may answer, “I know how to play football,” while the other answers, “I know your name.” Children with an understanding of the maxim of relation would choose the latter puppet.

### Controversial aspects of the CVT

This CVT task has primarily been used over the last decade to examine the awareness of Gricean maxim violations in atypical children (Surian et al., 1996, 2010; Surian and Siegal, 2001; Siegal et al., 2009, 2010). Although previous studies using the CVT have reported that children with rich access to language (e.g., bilingual children) outperformed other children (e.g., monolingual children) (Siegal et al., 2009, 2010), there is no clear evidence regarding the age at which typically developing children show awareness of Gricean maxim violations. Thus, the existing literature has failed to identify the developmental stages associated with the awareness of maxim violations.

To the best of our knowledge, the CVT is the only paradigm that has been used repeatedly in different countries; however, these studies have yielded inconsistent results. In studies conducted in England, Italy, Slovenia, and Japan, some children appeared to have difficulty detecting violations of either the first or second maxims of quantity, or both (Surian et al., 1996, 2010; Siegal et al., 2009, 2010). Moreover, Japanese monolinguals and Japanese-English bilinguals performed poorly on the maxim of quality (Siegal et al., 2010). There appear to be two possible reasons for the inconsistencies in the literature. One factor is cultural familiarity. Siegal et al. (2009) pointed out that children in different language groups might interpret some items differently due to different cultural experiences. When tested children in different language groups, Siegal et al. (2010) made minor changes to the original English-language version of the CVT to ensure cultural familiarity prior to testing children with Italian and Japanese monolinguals or Japanese-English bilinguals, but they did not test the suitability of these changes. Therefore, they may have included inappropriate items; this may have been especially true for the Japanese version of the CVT (e.g., they used “Japanese class,” *kokugo*, as one of their items, but Japanese children usually learn this only after starting school). This possibility could be resolved by testing adults within the same culture and selecting appropriate items prior to testing children.

The second factor is tone of voice, which has not been adequately addressed in previous studies; for example, this was not controlled similarly in English and Japanese stimuli (Siegal et al., 2010). Vocal affect is an important clue in understanding speakers' intentions. Four-year-olds possess this sensitivity and can use it to clarify a speaker's intended meaning (Berman et al., 2010). Thus, possible effects of tone of voice should be avoided, especially when children in different countries are tested and the results compared.

### Importance of the maxim of manner

Siegal (2008) suggested that the maxims of manner, along with the maxim of relation, may influence preschoolers' performance on an appearance-reality distinction task that asks two similar questions, each requiring a different answer (e.g., Flavell et al., 1983). In this task, an experimenter shows a child some objects that have two properties (e.g., a rock-like sponge) and asks the child two appearance and reality questions, such as “does it look like a rock or does it look like a sponge?” and “Is this really a rock or is it really a sponge?” Siegal (2008) suggested that it may be difficult for young children to correctly understand the similar but different questions and fail this task; in other words, they may not be able to distinguish obscure differences in the two questions. If they interpret this way, the experimenter's questions can violate the maxim of manner. Thus, attempts to understand preschoolers' comprehension ability should not ignore the maxims of manner.

### Purpose of the present study

We revised the CVT used in previous studies (Surian et al., 1996, 2010; Siegal et al., 2009, 2010) in several ways. First, we examined the maxim of manner, widely interpreted as “be perspicuous,” by selecting the second maxim of manner, which states that a respondent should avoid an ambiguous answer. Second, we tested adult samples and selected reliable and valid items for the revised CVT (CVT-R). Third, we used a neutral tone of voice for the stimuli. We then examined awareness of Gricean maxim violations in 4–6-year-olds and adults, to compare maxim understanding across age. We hypothesized that children and adults would be most aware of the violations of the maxim of relation. We also hypothesized that the detection of violation of any maxim would be especially difficult for 4-year-old children, because both monolingual and bilingual 4-year-olds have been found to exhibit less understanding of all maxims than older children (Siegal et al., 2009). Moreover, children aged five or older who have an understanding of theory of mind (Perner et al., 1987; Wellman et al., 2001) may begin to show an understanding of pragmatics, because these two abilities are closely related (Happe, 1993).

## Method

### Participants

Participants included 21 adults (*M* = 25.2 years, SD = 4.23; 10 males) and 64 preschoolers: 18 four-year-olds (*M* = 51.44 months, *SD* = 3.50, range = 47–59 months; 8 males), 23 five-year-olds (*M* = 65.2 months, *SD* = 3.77, range = 60–71 months; 12 males), and 21 six-year-olds (*M* = 75.7 months, *SD* = 2.75, range = 72–81 months; 10 males). Children were recruited from kindergartens and nursery schools in Hyogo and Osaka prefectures. Data from one of the 4-year-olds were excluded from analysis because of experimental error, and data from a 6-year-old who refused to complete the task were also excluded. Therefore, data from 62 of the 64 preschoolers were included in the analyses.

The design and purpose of the study were explained to the head administrators of the kindergartens and nursery schools, and their permission was obtained orally. Oral permission was also obtained from adult participants after they were informed of the design and purpose of the study. The study was conducted in accordance with the ethics principles of the Japanese Psychological Association, and the study design was approved by the ethics review board of Kobe University.

### Materials

CVTs used in previous studies had either 25 items (five questions for five maxims: the first and second maxims of quantity, the first maxim of quality, and the maxims of relation and politeness; (Surian et al., 1996; Siegal et al., 2009) or 20 items, including those mentioned above but excluding either the first maxim of quantity (Siegal et al., 2010) or the maxim of politeness (Surian et al., 2010). As noted above, previous studies have not examined the maxims of manner (“avoid obscurity of expression,” “avoid ambiguity,” “be brief,” and “be orderly”). In this study, we added conversations such as the following to examine the second maxim of manner:

Question: “Which do you like, tea or milk?”

Answer: “Maybe tea or maybe milk.” (The alternative, appropriate answer was “I like milk.”)

In the validation process, 30 adults (*M* = 24.7 years, *SD* = 6.45; 16 males) completed a questionnaire version of the CVT (CVT-Q) that required them to choose the answer that they considered wrong, silly, or rude. The CVT-Q consisted of 34 items and was based on the Japanese CVT used by Siegal et al. (2010). Although Siegal et al. made minor changes to account for cultural familiarity, a few questions and answers required further revision. For example, the question “What did you do at school?” with the answer “We did some writing” was not appropriate. Japanese schools call writing classes “Japanese” (*kokugo*), and *kokugo* classes are not taken until after children have completed their preschool education. By contrast, there are widely known school activities, such as those related to physical activity, that preschoolers can understand. Therefore, we changed the answer to “We played football.”

Based on the preliminary test, we excluded items with lower scores, and we selected 30 items (five questions for each maxim) for the Japanese CVT-R (see Appendix in Supplementary Materials for the list of items). We then created a video puppet play, which was similar in structure to those used in previous studies (Surian et al., 1996, 2010; Siegal et al., 2009, 2010). Specifically, three puppets were used to simulate short conversations (30 trials). One boy puppet asked questions of two girl puppets. One of the respondent puppets gave an answer that violated one of the maxims and the other respondent puppet gave an appropriate answer. Each of the puppets was dressed in clothes of a different color (blue, red, or yellow). To prevent preferences or biases for a particular puppet, the two respondent puppets randomly gave either appropriate or inappropriate answers. All conversations were recorded in a neutral tone of voice to eliminate clues other than those included in the conversational scripts.

### Procedure

Children and adults participated in the experiment individually. They were shown the puppet play video and asked to identify the puppets that gave the silly or rude answers. Two trial order conditions were used to prevent order effects: Approximately half of the children watched the video in one order (order 1) and the other half watched the video in the reverse of that order (order 2). All of the adult participants watched the video in order 1.

## Results

Table 1 shows the mean number of correct responses on the CVT-R for adults and children in the three age groups (score range 1–5). A preliminary 2 (gender: male, female) × 4 (age: 4 years, 5 years, 6 years, adult) × 6 (maxim: first and second quantity, quality, relation, politeness, and manner) mixed-design analysis of variance (ANOVA) was conducted to evaluate gender differences. Gender and age were between-subjects variables and maxim was within-subjects. The main effect of gender was not significant, *F*_(1, 73)_ = 1.87, *p* = 0.18, η ^2^_p_ = 0.03; however, the interaction between gender and age was marginally significant, *F*_(3, 77)_ = 2.25, *p* = 0.09, η ^2^*_p_* = 0.09. A simple main effect analysis suggested a gender difference only in 4-year-olds: Girls produced a higher number of correct scores than did boys (boys: 2.19, girls: 2.94). Since this was a marginally significant interaction, we combined males and females in further analyses. We also conducted a 2 (order: standard, reverse) × 3 (age: 4 years, 5 years, 6 years) × 6 (maxim: first and second quantity, quality, relation, politeness, manner) mixed-design analysis of variance (ANOVA) to evaluate order effects. Order and age were between-subjects variables and maxim was within-subjects. The main effect of order was not significant, *F*_(1, 52)_ = 0.79, *p* = 0.38, η^2^*_p_* = 0.02, and no interactions with order were significant. Therefore, no order effect was observed.

**Table 1:** **Mean numbers of correct responses to CVT and results of one-sample *t*-tests for all age groups**.

**Age groups**	**Maxims**	**Mean correct response scores (SD)**	**Results of one sample *t*-tests**
4-year-olds	Quantity I	2.88 (1.54)	*t*_(16)_ = 1.03
	Quantity II	2.06 (1.34)	*t*_(16)_ = −1.35
	Quality	2.65 (1.06)	*t*_(16)_ = 0.57
	Relation	2.82 (1.81)	*t*_(16)_ = 0.74
	Politeness	2.59 (1.12)	*t*_(16)_ = 0.32
	Manner	2.53 (1.62)	*t*_(16)_ = 0.08
5-year-olds	Quantity I	3.83 (1.34)	*t*_(22)_ = 4.76^**^
	Quantity II	2.61 (1.31)	*t*_(22)_ = 0.40
	Quality	4.13 (0.81)	*t*_(22)_ = 9.60^**^
	Relation	4.30 (1.11)	*t*_(22)_ = 7.83^**^
	Politeness	3.00 (1.48)	*t*_(22)_ = 1.62
	Manner	3.35 (1.30)	*t*_(22)_ = 3.13^**^
6-year-olds	Quantity I	4.05 (0.10)	*t*_(22)_ = 6.94^**^
	Quantity II	3.20 (1.01)	*t*_(22)_ = 3.11^**^
	Quality	4.45 (1.00)	*t*_(22)_ = 8.73^**^
	Relation	4.75 (0.55)	*t*_(22)_ = 18.29^**^
	Politeness	3.90 (1.02)	*t*_(22)_ = 6.13^**^
	Manner	4.00 (1.21)	*t*_(22)_ = 5.53^**^
Adults	Quantity I	4.86 (0.35)	*t*_(20)_ = 30.13^**^
	Quantity II	4.62 (0.59)	*t*_(20)_ = 16.47^**^
	Quality	4.90 (0.30)	*t*_(20)_ = 36.64^**^
	Relation	4.90 (0.30)	*t*_(20)_ = 36.64^**^
	Politeness	3.62 (1.02)	*t*_(20)_ = 5.01^*^
	Manner	4.90 (0.30)	*t*_(20)_ = 36.64^**^

* *p < 0.05*;

** *p < 0.01*.

A 4 (age: 4 years, 5 years, 6 years, adult) × 6 (maxim: first and second quantity, quality, relation, politeness, manner) mixed ANOVA with age as between-subjects and maxim as within-subjects variables revealed a significant main effect of age, *F*_(3, 77)_ = 35.76, *p* < 0.01, η^2^*_p_* = 0.58. Tukey HSD tests revealed that the mean response score for each age group differed significantly from those of the other groups. Moreover, the response scores increased with age. The main effect of maxim was also significant, *F*_(5, 385)_ = 15.30, *p* < 0.01, η^2^*_p_* = 0.17. *Post-hoc* Bonferroni tests indicated that children and adults were less sensitive to violations of politeness and second quantity than they were to violations of the other maxims. Scores for politeness were significantly lower than those for relation, quality (*p*s < 0.01), and first quantity (*p* < 0.05). Scores for the second quantity were significantly lower than those for relation, quality, manner, and first quantity (*p*s < 0.01). Overall, children's understanding was better for the maxims of relation and quality than for the other maxims. Scores for relation were significantly higher than those for politeness, second quantity (*p*s < 0.01), and manner (*p* < 0.05). Scores for quality were significantly higher than those for politeness and second quantity (*p*s < 0.01). In addition, scores for the first quantity were significantly higher than those for politeness (*p* < 0.05) and second quantity (*p* < 0.01).

The interaction of maxim and age was also significant, *F*_(15, 385)_ = 2.41, *p* < 0.01, η^2^*_p_* = 0.09. An analysis of simple main effects revealed that 5-year-olds' scores for the maxim of relation were significantly higher than those for politeness (*p* < 0.01), manner (*p* < 0.05), and second quantity (*p* < 0.01). Moreover, their scores for the maxim of quality were significantly higher than those for politeness (*p* < 0.01) and second quantity (*p* < 0.01); their scores for the first quantity maxim were significantly higher than those for second quantity (*p* < 0.01). Six-year-olds' scores for the maxims of relation and quality were significantly higher than those for second quantity (*p* < 0.01). There were no significant differences among the scores of 4-year-olds. Finally, adults' scores for the maxim of politeness were significantly lower than those for relation (*p* < 0.01), quality (*p* < 0.01), manner (*p* < 0.01), and first quantity (*p* < 0.05).

To ascertain whether adults and children of different ages were able to understand each maxim, one-sample *t-*tests were conducted to examine whether the means of correct CVT-R responses for each group were at or above chance levels (i.e., a score of 2.5; see Table 1). For all maxims, 4-year-olds' response scores were similar to levels that could have occurred by chance. Five-year-olds' response scores were above chance levels for the first maxim of quantity, and the maxims of quality, relation, and manner. All response scores for 6-year-olds and adults were above chance levels (see Table 1 for all *t*-test results).

## Discussion

The present study examined developmental changes in the awareness of Gricean maxim violations in 4–6-year-old typically developing Japanese children; similarities between children and adults were also studied. Our results confirmed that the maxim of relation is the easiest maxim to understand; this is consistent with the results of Eskritt et al. (2008). Children exhibited better performance when detecting violations of the maxim of quality, which is partially consistent with the findings of Vázquez et al. (2013). Moreover, Japanese children had the most difficulty detecting violations of the second maxim of quantity (i.e., providing more information than required information). While Siegal et al. (2010) found that Japanese children performed poorly in the identification of violations of the maxim of quality, we did not replicate this result after conducting a preliminary screening of the relevance of the measure in a group of adults. Therefore, the cultural differences reported by Siegal et al. (2010) may have been an artifact of inappropriate stimuli. The neutral tone of voice used in the CVT-R in the present study may also have helped to eliminate inconsistent results between previous studies. Furthermore, children showed sensitivity to violations of the second maxim of manner (i.e., avoid ambiguity) by the age of 5 years. Finally, we found that children and adults evaluated the maxim of politeness differently.

The pattern of results differed with age group. As we hypothesized, 4-year-olds did not correctly identify the puppet that violated Gricean maxims. Their comprehension was significantly lower than that of 5-year-olds, 6-year-olds, and adults; furthermore, their scores were at a level that could have occurred by chance. However, this result does not indicate that 4-year-olds have no understanding of Gricean maxims. Four-year-olds may be able to demonstrate their awareness of Gricean maxims implicitly, which may be the reason they were able to perform adequately on game-based tasks (e.g., selective-trust tasks), for example, when they received stickers if they won the games (Eskritt et al., 2008) or were required to learn a new word from a trusted partner (Vázquez et al., 2013). These methods might help 4-year-olds concentrate on the tasks, improving their performance. In addition, the CVT-R requires greater metacognitive abilities or cognitive load, as it asks children to choose the “rude” or “silly” answer from two options. Therefore, children may need to inhibit the selection of a puppet that gives a preferred response. Thus, if children are asked to choose the puppet that gives the correct response, 4-year-olds may be more likely to respond appropriately.

Five-year-olds understood only some of the maxims; thus, they appeared to be in a transitional period. This may indicate that children acquire the understanding of theory of mind before they start to understand pragmatics. Interestingly, 5-year-olds were able to detect maxim of manner violations; thus, this maxim may be easier to understand than the other maxims (i.e., the second maxim of quantity and the maxim of politeness).

Six-year-olds and adults correctly selected the violations for all of the Gricean maxims tested. This indicates that children demonstrate acquisition of adult-level awareness of Gricean maxim violations by the age of 6 years, when this is measured via CVT. Previous studies examining maxim violation awareness via children's evaluations of others' conversations concluded that this ability was not fully developed until children had reached the school-age years (e.g., Conti and Camras, 1984; Vázquez et al., 2013); however, the present study showed awareness of Gricean maxim violations in younger children when this was examined via more simple and controlled stimuli and included all six maxims.

Interestingly, adults' performance on the maxim of politeness was lower than their performance on other maxims, with the exception of the second maxim of quantity. Six-year-olds did not show this tendency. At the end of each trial, we asked the children why they had thought that their chosen puppet had said something silly or rude; not all children answered this question, which is consistent with research indicating that young children do not answer open-ended questions adequately (Ceci and Bruck, 1993, 1995; Peterson and Bell, 1996; Fivush et al., 2002). However, some of the children reported that their teachers had told them not to say anything rude or impolite. More specifically, they said it was cruel to say something rude to others (e.g., to friends). In contrast, adults reported that it would be kinder to tell the truth (e.g., if a friend is wearing inappropriate clothing, saying “I don't like it” is preferable to saying “yes they are very nice”). Therefore, the present results suggest that the children judged the maxim of politeness based on the principles of morality learned in school, while adults' judgments were more complex.

In addition, we found that 4-year-old girls tended to show better performance on the CVT than boys of the same age. Some studies have suggested that girls develop language skills more rapidly than boys. However, the results regarding the emergence of this advantage are mixed. Some studies have indicated that the advantage occurs before age of three (e.g., Eriksson et al., 2012) and others that it occurs after the age of 11 (e.g., Hyde and Linn, 1988). Although the gender difference might increase with age, our results did not follow this pattern. Eriksson et al. (2012) tested over 13,000 European children who spoke 10 different languages and found robust advantages for girls. Our sample was limited and our findings did not show this trend, so it would be premature for us to draw any conclusion about gender differences on the basis of the present results.

In the present study, developmental patterns of understanding Gricean maxims in childhood and adulthood were examined. Violations of the maxim of relation were shown to be the easiest to detect (see also Sperber and Wilson, 1986/1995; Surian et al., 1996, 2010; Eskritt et al., 2008). Because 4-year-olds are unlikely to notice others' violations of Gricean maxims, researchers should pay special attention to their responses when testing young children in developmental psychology experiments. Researchers should also remember that older preschoolers who respond similarly to adults may nevertheless interpret task materials in a different manner. That is, the fact that preschoolers have an understanding of the maxim of politeness that is different from that of adults should be considered when verbal tasks are developed for use in developmental research. The maxim of politeness is unique in its flexibility, and its standards may change throughout life.

Finally, the CVT has its limitations. Although the lengths of violating and conforming responses were controlled, it was difficult to control the degree of obviousness of all items. Also, the CVT asked children to choose “silly” or “rude” responses, as in other experimental studies of Gricean maxims. Although this response requirement may not exactly follow Grice's cooperative principle, it is the easiest way for children to show their understanding of Gricean maxim violations empirically. This study represents a first step in understanding how pragmatic ability matures in typically developing preschoolers.

### Conflict of interest statement

The authors declare that the research was conducted in the absence of any commercial or financial relationships that could be construed as a potential conflict of interest.
