# Supplementary material for: Understanding violations of Gricean maxims in preschoolers and adults
Source: Front Psychol. 2015 Jul 2;6:901. doi: 10.3389/fpsyg.2015.00901 (PMC4488609; doi:10.3389/fpsyg.2015.00901)
Supplement: Supplementary file 1 [file DataSheet1.DOCX]

Appendix

| Maxims | Questions |  | Puppet 1 | Puppet 2 |
| --- | --- | --- | --- | --- |
| Relation | What sports do you know? |  | I know how to play baseball. | I know your name. |
|  | What did you do at school? |  | We took a bath. | We played football. |
|  | What did you do on the summer holidays? |  | I cycled every day. | My dress is yellow. |
|  | What is your favorite program on television? |  | I like Anime. | I like sandwiches. |
|  | What do you like to eat? |  | I like Kyoto. | I like ice cream. |
| Quality | Where do you live? |  | I live on the moon. | I live in Tokyo. |
|  | Is there any more chocolate? |  | Yes, I am made of chocolate. | Yes, I saved you a piece. |
|  | What color are apples? |  | Red. | Black. |
|  | Do you have any brothers? |  | Yes, I have 500. | Yes, I have two. |
|  | Have you seen my dog? |  | Yes, he was in the clouds. | Yes, he was in the garden. |
| Quantity I | What did you see at the cinema yesterday? |  | Snow White. | A movie. |
|  | What did you eat for lunch? |  | I had some food. | I had Ramen noodles. |
|  | What kind of juice do you like? |  | Apple juice. | Juice in a cup. |
|  | What did you get for your birthday? |  | A present. | A bike. |
|  | What would you like to buy in the toy shop? |  | A toy. | A video game. |
| Quantity II | What pets do you like? |  | I like puppies. | I like rabbits because they have four legs and a tail. |
|  | Where did you go this morning? |  | I went to my grandmother's place and I had a great time. | I went to my friend's place and it was a house. |
|  | What is your favorite color? |  | Yellow, which is a color. | Blue like the sea. |
|  | Who is your best friend? |  | My best friend is Ken. He wears trousers. | My best friend is Yosiko. She goes to my school. |
|  | What did you have for breakfast? |  | I had a boiled egg and toast. | A hardboiled egg cooked in hot water in a small sauce pan. |
| Politeness | Do you like my clothes? |  | Yes, they are very nice. | No, I don’t like them. |
|  | Would you like some of my cake? |  | Yes, thank you. | No, it does not look good. |
|  | Could you help me in tidying up my room? |  | No, your room is dirty. | I have something to do now and I cannot. |
|  | May I borrow your pencils? |  | No, you are bad at drawing. | No, I left them at home. |
|  | Do you want to play with me? |  | No, it is boring to play with you. | No, sorry, I am too tired to play now. |
| Manner | Have you ever taken a train? |  | Yes. It was so fast. | I may or may not have. |
|  | Which do you like, tea or milk? |  | Maybe tea, or maybe milk. | I like milk. |
|  | With whom will you play today? |  | Um, I am not sure. | I will play with Yuko. |
|  | How old are you? |  | Maybe about 7 years old. | 8 years old. |
|  | Did you have a snack? |  | Maybe I had some chocolate. | I had a doughnut. |
